# An Altered Peptide Ligand derived from heat-shock protein 60 induces regulatory T cells in JIA patients and suppresses adjuvant-induced arthritis

**DOI:** 10.1186/1546-0096-9-S1-P182

**Published:** 2011-09-14

**Authors:** Dolores Cantera, Noraylis Lorenzo, Ariana Barberá, Amarys Alonso, Yamile Heredia, Elsy Chall, Lourdes Franco, Ramón G Padrón, Maria C Domínguez

**Affiliations:** 1 Pedro Borrás Hospital, Havana Cuba; 2 Center for Engineering Genetic and Bioctenology, Havana Cuba; 3 Willian Soler Hospital, Havana Cuba; 4 Pediatric Hospital of Centro Havana, Cuba

## Background

Induction of immune tolerance as therapeutic approach for autoimmune diseases constitutes a current research focal point. JIA is the most common chronic rheumatic disorder in children and is also a major cause of acquired disability and impairment of quality of life in childhood.

## Aim

In this sense, we aimed to evaluate an Altered Peptide Ligand (APL) for induction of peripheral tolerance in patients with Juvenile idiopathic Arthritis (JIA).

## Methods

First, a novel T cell epitope from human heat-shock protein 60 (Hsp60), an autoantigen involved in the pathogenesis of JIA and other autoimmune diseases, was identified by bioinformatics tools and an APL was design starting from this epitope. We evaluated the therapeutic effect of this peptide in an adjuvant-induced arthritis (AA) rat model. Clinical score, TNFα levels and histopathology were monitored. Also the potentialities of the APL for inducing regulatory T cells were evaluated in ex vivo assays using peripheral blood mononuclear cells (PBMC) from JIA patients.

## Results

The APL efficiently inhibited the course of AA in rat, with significant reduction of the clinical and histopathogy score. This effect was associated with a decrease of TNFα levels in spleen. Finally, stimulation of PBMCs from JIA patients by the APL increases the proportions of the CD4+CD25^high^ FoxP3+ Treg cells.

## Conclusion

These results indicate a therapeutic potentiality of APL and support further investigation of this candidate drug for treatment of JIA and other autoimmune diseases.

